# Hybrid Approach for Indoor Localization Using Received Signal Strength of Dual-Band Wi-Fi

**DOI:** 10.3390/s21165583

**Published:** 2021-08-19

**Authors:** Byeong-ho Lee, Kyoung-Min Park, Yong-Hwa Kim, Seong-Cheol Kim

**Affiliations:** 1 Department of Electrical and Computer Engineering, Institute of New Media & Communications, Seoul National University (SNU), Seoul 08826, Korea; bhlee@maxwell.snu.ac.kr (B.-h.L.); rudals319@maxwell.snu.ac.kr (K.-M.P.); 2 Department of Data Science, Korea National University of Transportation, Uiwang-si 16106, Korea; yongkim@ut.ac.kr

**Keywords:** dual-band, indoor localization, range-based localization, received signal strength, trilateration, Wi-Fi

## Abstract

In this paper, we propose a hybrid localization algorithm to boost the accuracy of range-based localization by improving the ranging accuracy under indoor non-line-of-sight (NLOS) conditions. We replaced the ranging part of the rule-based localization method with a deep regression model that uses data-driven learning with dual-band received signal strength (RSS). The ranging error caused by the NLOS conditions was effectively reduced by using the deep regression method. As a consequence, the positioning error could be reduced under NLOS conditions. The performance of the proposed method was verified through a ray-tracing-based simulation for indoor spaces. The proposed scheme showed a reduction in the positioning error of at least 22.3% in terms of the median root mean square error compared to the existing methods. In addition, we verified that the proposed method was robust to changes in the indoor structure.

## 1. Introduction

Indoor localization technology is indispensable for Internet of Things services. It can be applied to a wide range of fields, such as navigation for smartphone users, resource management in smart factories, and autonomous driving of robots, among others. Outdoors, the global navigation satellite system (GNSS) is available to provide position information. However, the GNSS is inappropriate indoors owing to the issue of the blocking of the signals from satellites. Several studies have been conducted in order to estimate locations in GNSS-denied environments. In particular, the method of using the received signal strength (RSS) from Wi-Fi communication is promising because it fits most commercial products [1,2,3].

Indoor localization methods that use RSS can be divided into two groups: fingerprint-based methods and range-based methods [2]. The fingerprint-based methods are based on a pattern-matching scheme. The RSS patterns from multiple access points (APs) at a specific location were recorded in the offline stage. In the online stage, the measured RSS pattern is compared with a recorded database to match a location. The offline stage requires time and effort for site surveys and is vulnerable to channel changes (e.g., AP location changes and furniture structure changes) [3]. Range-based localization methods estimates the distance between individual APs and a target node and specify a location through geometric inference. The main problem in range-based localization using RSS is the ranging error. The RSS ranging error result from the non-line-of-sight (NLOS) channel condition, antenna direction, and time-varying obstruction by crowds. Among these, the NLOS effect of indoor wall structures and obstacles is the most important factor. The shadowing effect caused by wall structures makes RSS ranging difficult, resulting in position inaccuracy [4,5].

Therefore, improving the ranging accuracy is a key factor for range-based localization. Typically, RSS ranging is based on a path-loss model (PLM), which represents the relation between the RSS and the distance between communication nodes. The representative model is the two-slope model presented in the IEEE 802.11 standard [6]. This is an expression for an universal indoor space and does not reflect a site-specific environment. Therefore, the ranging accuracy based on this model is limited in practical cases. To solve this problem, studies have been conducted on the adjustment of the parameters of the PLM [7,8,9,10] and the correction of ranging results through the detection of NLOS by using RSS changes over time or characteristics of differences in RSS in dual-frequency bands.

The method of adjusting the PLM’s parameters to fit into a specific environment improves the statistical ranging accuracy; however, there is a limit to reflecting complex indoor spaces with severe NLOS conditions. The method that uses the time-varying RSS has a disadvantage in terms of time consumption. The dual-band scheme is based on the wave propagation characteristics in an NLOS environment with a carrier frequency. In practice, many off-the-shelf Wi-Fi APs and mobile devices are compatible with dual-band operation.

In addition, many approaches use deep learning techniques. Most localization algorithms that use deep learning can be regarded as data-driven methods, as they involve a neural network model that is trained with RSS input and the corresponding position labels. Replacing the entire localization process with a deep learning model causes problems regarding vulnerability to environmental changes, similarly to fingerprint-based methods. In this study, we propose a hybrid approach, which applies data-driven deep learning to only the ranging part of a model-based localization algorithm. By performing nonlinear regression using a neural network for the dual-band RSS, the ranging accuracy was significantly improved compared to that of the rule-based method. In addition, the proposed localization method has the advantage of being robust against changes in the AP locations and spatial structure. A ray-tracing method was used to get the channel data for a complex NLOS environment, and the ranging performance of the proposed dual-band RSS was evaluated and compared to that of existing methods. A comparison with existing methods using single-band RSS or rule-based ranging showed the advantages of the proposed method in terms of positioning accuracy. In addition, changes in the neural network structure of the deep regression model were analyzed.

The remainder of this paper is organized as follows. In the next section, we introduce some related studies. Section 3 explains the background of the propagation characteristics of electromagnetic waves indoors. Section 4 presents the system model and the proposed hybrid localization method using dual-band RSS. Section 5 presents a performance analysis of the proposed method compared to existing methods, and Section 6 outlines the conclusions of the study.

## 2. Related Works

For indoor environments, in which it is difficult to use GNSS, there have been studies that have used various alternative systems to find the locations of devices. There are infrastructure-less methods that avoid dependence on a specific infrastructure by measuring a passive signal with a magnetometer, an inertial sensor, a microphone, or a light sensor [11,12,13,14]. On the other hand, some localization methods depend on the infrastructure, which takes over the role of the satellites in the GNSS. Some studies used an ultra-wide-band tag [15], Bluetooth beacon [16,17], or radio frequency identification tag [18]. Because these methods have weaker signal strength and smaller coverage than Wi-Fi systems in indoor environment with many walls. In addition, they require a deployment of the infrastructure. Although localization methods that use Wi-Fi also require the placement of Wi-Fi APs, many Wi-Fi APs are already currently deployed. Therefore, localization methods that use Wi-Fi have the advantage of being used without the installation of additional infrastructure compared to other infrastructure-based methods.

Especially for smartphones, studies combining Wi-Fi and the other infrastructure-less methods have been conducted. Several state-of-art approaches used a fine timing measurement (FTM) protocol supported by a Wi-Fi system, which is a method of using a measurement of the distance based on the round-trip time by exchanging packets when both the AP and the device support the FTM protocol [19]. In particular, such approaches showed high ranging accuracy under LOS conditions [20,21], and these studies using a fusion of FTM and other sensors showed high accuracy in tracking the path of a pedestrian [22,23,24].

To reduce the dependency on infrastructure as much as possible, there are methods that use the RSS of a Wi-Fi beacon’s signal. Wi-Fi RSS depends on the indoor wave propagation characteristics, and especially the path-loss properties with respect to the distance. Many studies have been conducted on indoor path loss, which has been integrated into statistical models [6,25,26]. The ITU-1238 model in [25] assumes the PLM in the indoor environment as a 1-slope model, and presents path loss parameters for several open space and NLOS environments. The models introduced in [6,26] are based on 2-slope model, considering the complex indoor environment. However, these channel models were used to analyze the quality of service in communication. Range-based localization requires site-specific channel information. In particular, a detailed model of the shadowing effect caused by walls is necessary. In [27], the propagation loss for a common building material in the 2.4 and 5.2 GHz bands was analyzed. In [28], the effects of walls on the propagation loss in complex indoor environments were analyzed, and an effective wall loss model was proposed.

Meanwhile, most indoor localization studies that use RSS rely on a fingerprint-based method. Existing single-band based fingerprinting techniques [3,29,30,31] demonstrate good localization accuracy, but require a thorough site survey of the target space. Channel state information (CSI) was also measured in [29,30,31]. Fingerprinting methods that use CSI provide high accuracy. However, there is a risk that they may not be robust to AP location changes after a site survey because CSI is sensitive to spatial configurations. In addition, CSI has a high data overhead and can only be acquired with specific devices.

Some studies used dual-band RSS for fingerprint-based positioning [32,33,34,35,36,37]. Consequently, the accuracy of these methods depends on site surveys, and vulnerable to environmental changes. In [34], rule-based localization was exploited by using dual-band RSS with consideration of NLOS conditions. The channel conditions were evaluated by comparing the RSS attenuation between the two bands, and different ranging functions were applied according to the channel conditions. However, the authors of [34] classified the channel conditions into three types, which were insufficient to reflect indoor channel conditions. The use of deep learning has been attempted in order to overcome the limitations of rule-based methods. The authors of [38,39,40] estimated positions by training a neural network with RSS vectors from multiple APs. These were types of fingerprint-based methods that derived the location coordinates output from the neural network, which was trained with RSS patterns. Therefore, similarly to other fingerprint-based methods, there was a disadvantage in that they could only operate in a pre-surveyed environment.

Previous studies about indoor localization using Wi-Fi RSS with deep learning techniques were mainly based on fingerprinting. The fingerprint-based methods can achieve high positioning accuracy, but require heavy site surveys and are vulnerable to minor environmental changes, such as changes in APs’ locations or obstructions. For these reasons, the goal of this study is to present a range-based localization method that is robust to changes in APs and that minimizes the need for site survey. The range-based method using only RSS has challenges because of the ranging error in the indoor environment with many walls. The model based ranging had a limitation in accurate ranging in the complex indoor environment. Therefore, we intended to improve the RSS ranging accuracy by applying data-driven deep learning to only the ranging part of the range-based localization algorithm, and evaluated the effect of the method on positioning accuracy in indoor environment.

## 3. Preliminary

**Figure 1:**
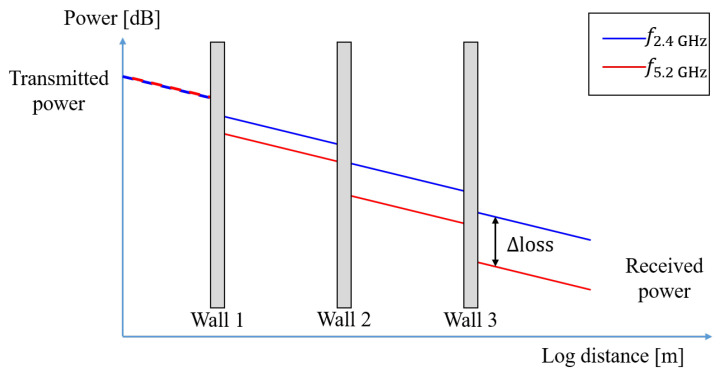
Indoor path loss for the different frequency bands.

A free-space PLM can be represented based on the Friis transmission formula [41].

(1) $${\mathrm{PL}}_{\mathrm{fs}}=20\log_{10}\left(d\right)+20\log_{10}\left(f\right)+20\log_{10}\left(\frac{4\pi }{c}\right)-G_{\mathrm{Tx}}-G_{\mathrm{Rx}},$$

where ${\mathrm{PL}}_{\mathrm{fs}}$ denotes the free-space loss on the decibel scale, *d* is the distance (m) between the transmitter and receiver, *c* is the speed of light, *f* is the frequency, and $G_{\mathrm{Tx}}$ and $G_{\mathrm{Rx}}$ are the antenna gains of the transmitter and receiver, respectively. The PLM in a typical LOS environment is similar to that in free space, and the path-loss difference between the 2.4 and 5.2 GHz bands only has an offset according to the antenna gain and the frequency difference. However, in an NLOS environment, the PLM becomes more complicated. The site-general models are presented in previous research for indoor environments, [6,25,26]. Among them, we consider the IEEE 802.11 specification [6], which suggest a 2-slope model for the indoor Wi-Fi systems. The general model can be represented as shown in (2).

(2) $$r\left(d\right)=\left\{\begin{array}{c} r\left(d_{0}\right)-10\eta_{0}\cdot \log_{10}\frac{d}{d_{0}}+X_{0},\;\mathrm{if}\;d\leq d_{BP}\\ r\left(d_{BP}\right)-10\eta_{1}\cdot \log_{10}\left(d-d_{BP}\right)+X_{1},\;\mathrm{if}\;d>d_{BP}, \end{array}\right.$$

where r(d) is the RSS at distance *d*, and $r(d_{0})$ is the RSS at the reference distance $d_{0}$. $d_{BP}$ is the break-point distance, which divides two regions according to the distance from the transmitter. The two regions are modeled using different path-loss exponents, which are denoted by $\eta_{0}$ and $\eta_{1}$, and different shadowing factors, which are denoted by $X_{0}$ and $X_{1}$, respectively. This model is not appropriate for positioning, as it is a statistical model for analyzing the quality of service in general indoor environments. When radio waves propagate in a space with obstacles, they are affected by various phenomena, such as reflection, scattering, and diffraction. As a result, the path loss in an NLOS environment becomes extremely complex, and the average attenuation depends on the material of the obstacle and the radio wave frequency. For example, a cement wall, which is the most popular type of wall, shows significant differences in transmission and reflection properties for the 2.4 and 5.2 GHz frequencies [27]. Considering the influence of walls, Obeidat et al. [28] presented the following effective wall loss model, which is consistent with practical indoor environments:

(3) $$r\left(d\right)=r\left(d_{0}\right)-10\eta_{f}\cdot \log_{10}\left(\frac{d}{d_{0}}\right)-\Sigma_{v=1}^{V}W_{v},$$

where $\eta_{f}$ is the path-loss exponent, and $W_{v}$ is the attenuation caused by the *v*-th wall in the propagation path. Focusing on the fact that the wall attenuation differs for each frequency band, the NLOS mode can be estimated. Figure 1 conceptually represents the attenuation tendency of the walls for two frequency bands. In free space, the attenuation in two bands is similar, but the difference in attenuation between two bands increases as there are more obstacles since the attenuation in 5.2 GHz is larger than that in 2.4 GHz for obstacles. In Figure 1, the path loss is expressed according to the number of walls as if the discrete steps are clearly separated; however, in a practical environment, this is not clear because the attenuation varies depending on the wall thickness, material, and map composition. The key point is that the attenuation in the 5.2 GHz band for the same geometric environment tends to be large at a certain rate compared to that in the 2.4 GHz band, and this can be an indicator of the severity of the NLOS condition. We assume that there is a nonlinear relation between the severity of the NLOS condition and the difference in attenuation between two bands.

## 4. System Model

**Figure 2:**
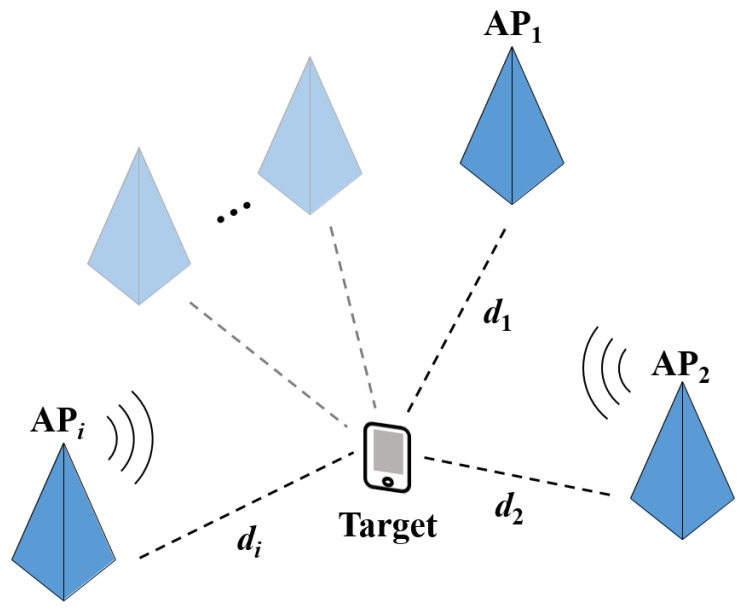
Range-based localization scenario.

In this study, we assumed a Wi-Fi communication environment that uses a dual-band frequency. As shown in Figure 2, when the RSS at the target point x$={\left[x,y\right]}^{T}$ is measured by three or more APs, the RSS of the *i*-th AP is represented as $r_{i}=\left[r_{i}^{2.4},r_{i}^{5.2}\right]$, where $r_{i}^{2.4}$ and $r_{i}^{5.2}$ denote the RSS of 2.4 GHz and that of 5.2 GHz, respectively.

**Figure 3:**
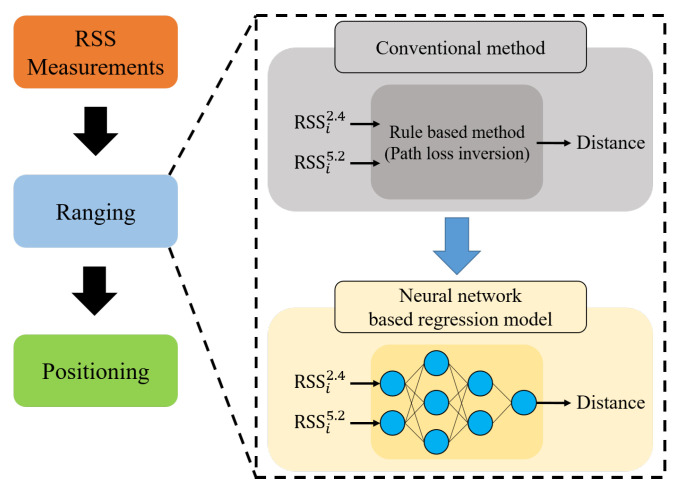
Proposed hybrid range-based localization scheme.

The proposed hybrid localization scheme is illustrated in Figure 3. The basic structure is the same as that of the typical range-based localization method. The distances between APs and target devices are estimated based on the RSS measured at the ranging stage, and the position of the target node is estimated using the *n*-distance estimates of *n* AP locations. In the ranging stage, the conventional rule-based method estimates the distance by using the inversion of the PLM, which represents the RSS attenuation according to the distance. For example, using the PLM in (2), the distance estimate $\hat{d}$ for the RSS value *r* can be calculated as follows.

(4) $$\hat{d}\left(r\right)=\left\{\begin{array}{c} d_{0}\cdot 10^{\frac{r(d_{0})-r}{10\eta_{0}}},\;\mathrm{if}\;r\leq r\left(d_{BP}\right)\\ d_{BP}+10^{\frac{r(d_{BP})-r}{10\eta_{1}}},\;\mathrm{otherwise}. \end{array}\right.$$

In this study, we replaced this ranging part with a regression model that used deep neural networks. The details of the proposed method of using a neural network are explained in the next section. After the distances from each AP are estimated, the positions of the target nodes are specified in the positioning stage. By using the known positions of the fixed APs and the estimated distances, trilateration-based algorithms can be applied. Among the various trilateration-based algorithms, the iterative least-square (ILS) method estimates the target position to be close to the optimal solution [42,43]. The position estimate using ILS can be expressed as follows:

(5) $${\hat{x}}_{}^{\left(k\right)}=\left[\begin{array}{c} {\hat{x}}^{\left(k\right)}\\ {\hat{y}}^{\left(k\right)} \end{array}\right]={\hat{x}}_{}^{\left(k-1\right)}+\left[\begin{array}{c} \delta x\\ \delta y \end{array}\right],$$

where

(6) $$\left[\begin{array}{c} \delta x\\ \delta y \end{array}\right]={\left[\begin{array}{ccc} \frac{\left(x_{1}-{\hat{x}}^{\left(k-1\right)}\right)}{d_{1}^{\left(k-1\right)}} & \; & \frac{\left(y_{1}-{\hat{y}}^{\left(k-1\right)}\right)}{d_{1}^{\left(k-1\right)}}\\ \vdots & \; & \vdots \\ \frac{\left(x_{N}-{\hat{x}}^{\left(k-1\right)}\right)}{d_{N}^{\left(k-1\right)}} & \; & \frac{\left(y_{N}-{\hat{y}}^{\left(k-1\right)}\right)}{d_{N}^{\left(k-1\right)}} \end{array}\right]}^{+}\left[\begin{array}{c} {\hat{d}}_{1}-d_{1}^{\left(k-1\right)}\\ \vdots \\ {\hat{d}}_{N}-d_{N}^{\left(k-1\right)} \end{array}\right],$$

and

(7) $$\begin{array}{c} d_{i}^{\left(k-1\right)}=\sqrt{{\left(x_{i}-x^{\left(k-1\right)}\right)}^{2}+{\left(y_{i}-y^{\left(k-1\right)}\right)}^{2}}, \end{array}$$

where, ${\hat{x}}_{}^{\left(k\right)}$ is the position estimate for the *k*-th iteration, and $\left(x_{i},y_{i}\right)$ is the position of the *i*-th AP. The initial position estimate ${\hat{x}}_{}^{\left(0\right)}$ can be set as the center of the map. The proposed scheme is a hybrid method that is similar to the existing rule-based method, but only the ranging part was replaced with the data-driven deep learning method. The risk of overfitting was minimized by the data-driven learning part. Ultimately, the position accuracy is increased by improving the ranging accuracy.

## 5. Proposed Ranging Method

**Figure 4:**
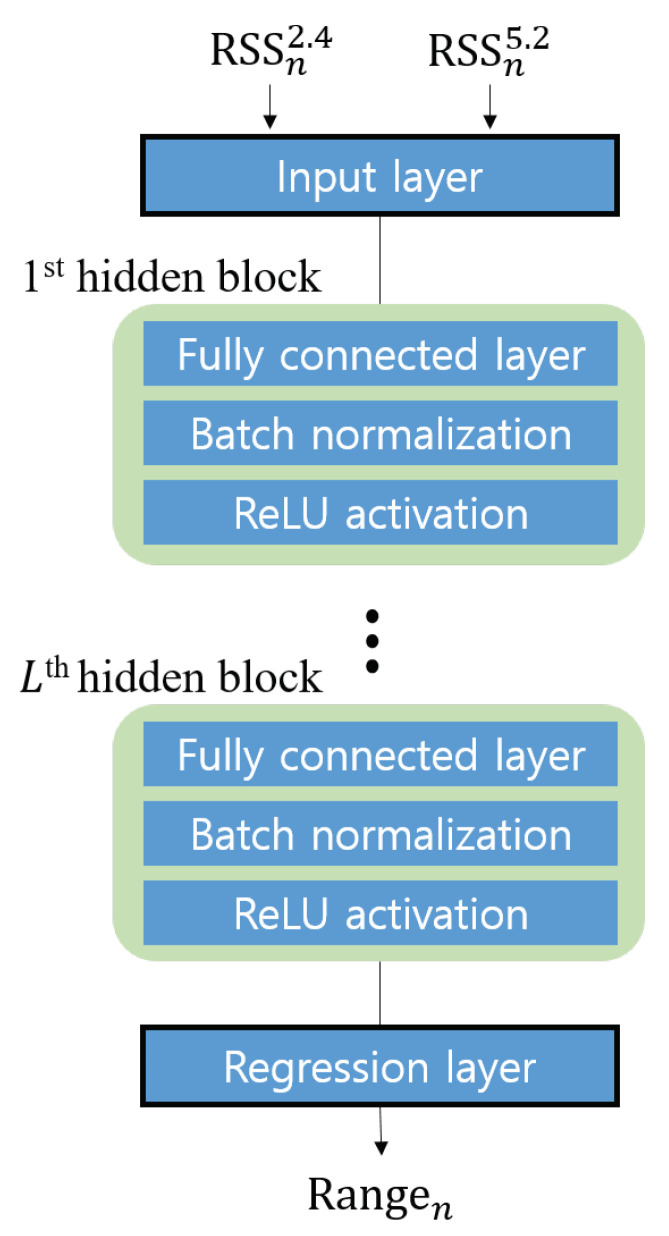
Neural network structure for deep regression.

In this study, we propose a deep regression model for dual-band RSS ranging. The model’s structure is shown in Figure 4. The input of the model is the dual-band RSS vector, $r_{i}$. The model has several hidden blocks, each of which is composed of a fully connected (FC) layer, a batch normalization layer, and a rectified linear unit (ReLU) activation function. The FC layer is a structure in which one node is connected to all other nodes in the adjacent layer. It allows for nonlinear regression along with a nonlinear activation function. Although the FC layer is simple and powerful, it is vulnerable to overfitting. To solve this problem, a batch normalization layer was inserted into each hidden block. The use of the batch normalization layer mitigates internal covariate shift phenomenon, which is the changes in the input data distribution affect the training procedure. Typically, batch normalization makes the neural network more stable and prevents the overfitting problem and increases the learning efficiency [44].

When the number of nodes of the FC layer of the *l*-th hidden block is $M^{\left(l\right)}$, an input is represented as $a_{l}={\left[a_{1},\ldots ,a_{M^{\left(l\right)}}\right]}^{T}$, and the output of the FC layer is expressed as follows:

(8) $$y^{\left(l\right)}=\left[\begin{array}{c} y_{1}^{\left(l\right)}\\ \vdots \\ y_{M}^{\left(l\right)} \end{array}\right]=W_{l}\cdot a_{l}+b_{l},$$

where $W_{l}$ is an $M^{\left(l-1\right)}\times M^{\left(l\right)}$ matrix representing the weights of the nodes in the FC layer, and $b_{l}$ is a vector representing biases. These are parameters that can be trained using data-driven learning. With the mini-batch size $K_{B}$, the batch normalization result ${\bar{y}}_{m}^{\left(l\right)}$ for the *j*-th sample is expressed as follows:

(9) $${\bar{y}}_{m}^{\left(l\right)}=\gamma_{m}^{\left(l\right)}\cdot (\frac{y_{m,j}^{\left(l\right)}-\mu_{m,B}}{\sqrt{\sigma_{m,B}^{2}+\epsilon }})+\beta_{m}^{\left(l\right)},$$

where $j\in [1,K_{B}]$, $y_{m,j}^{\left(l\right)}$ is the *m*-th node of the FC layer in the *l*-th hidden block. $\mu_{B}$ and $\sigma_{B}^{2}$ denote the sample mean and sample variance of $y_{m}^{\left(l\right)}$ in the mini-batch, respectively. The parameters $\gamma_{m}^{\left(l\right)}$ and $\beta_{m}^{\left(l\right)}$ are the scaling factors and shift factors, respectively, and these are also updated through the learning process. By re-centering and re-scaling the data for each layer, after batch normalization, the ReLU activation function is applied. The use of ReLU activation function mitigates the gradient vanishing problem that may occur in the FC layer, and allows the neural network with deep structure to be trained efficiently [45]. After the ReLU activation, the output of the *l*-th hidden block is as follows:

(10) $$o_{m}^{\left(l\right)}=\left\{\begin{array}{c} \;0,\;\;\;\;\;\;\;\;\;\;\;\;\mathrm{if}\;\;{\bar{y}}_{m}^{\left(l\right)}<0\\ {\bar{y}}_{m}^{\left(l\right)},\;\;\;\;\;\;\;\;\;\mathrm{otherwise} \end{array}\right.$$

The regression layer after the total of *L* hidden blocks is the FC layer with one node, which performs the linear combination of the *L*-th hidden block’s output. The proposed regression model can be trained with supervised learning by using the dual-band RSS vector and the ground truth distance value as a label. Let the ground truth distance be $d_{i}$, which corresponds to the *i*-th RSS vector; then, the mean square error (MSE) *E* with the model output can be expressed as follows:

(11) $$E=\sqrt{{\left(y_{i}-d_{i}\right)}^{2}}.$$

To train the proposed model, the gradient of the MSE in (11) with respect to each parameter is computed by using the back-propagation algorithm, and the parameters of the model are updated to minimize the average MSE for the training dataset.

The overall process of the proposed localization method is summarized in Algorithm 1.
**Algorithm 1** Proposed localization procedure.**<Offline stage>****Input**    Training set $R^{\prime }$: The set of dual-band RSS vector, $r_{i}=\left[r_{i}^{2.4},r_{i}^{5.2}\right]$,    and ground truth distance $d_{i}$ for each sample    Validation set $V^{\prime }$: The set of validation data, which are same kind of data as,    but not included in the training setInitialize a neural network described in Figure 4 with random weights, biases, and normalization parameters.**for** all training data grouped by mini-batch size of $K_{B}$, **do**    Forward calculation for the NN    The output $y_{i}$ is a result of the last layer of the NN    Calculate the average MSE between $y_{i}$ and $d_{i}$ in the mini-batch    Update parameters of the NN, by using back–propagation**end for**Find best–fit parameters of the NN that minimize MSE for the validation set**Output** Trained NN as a deep regression model **<Online stage>****Input**    Test data location of *N* APs and *N* RSS vectorSet a confidence threshold $Th_{c}$**for** each *i*-th AP **do**    Predict ${\hat{d}}_{i}$ using the trained NN**end for**Find target position $\hat{x}$, using Equations (5)–(7)**Output**: Position estimate of target device $\hat{x}={\left[\hat{x},\hat{y}\right]}^{T}$

The number of hidden blocks and the number of hidden nodes constituting the proposed model are user-defined hyperparameters. The deeper the neural network is, the more complex the nonlinear function will be. However, there is a performance limit when the structure is excessively complex compared to the input data. In the next section, an analysis of the performance depending on the hyperparameters is presented.

## 6. Performance Evaluation

### 6.1. Ray-Tracing-Based Simulation

**Figure 5:**
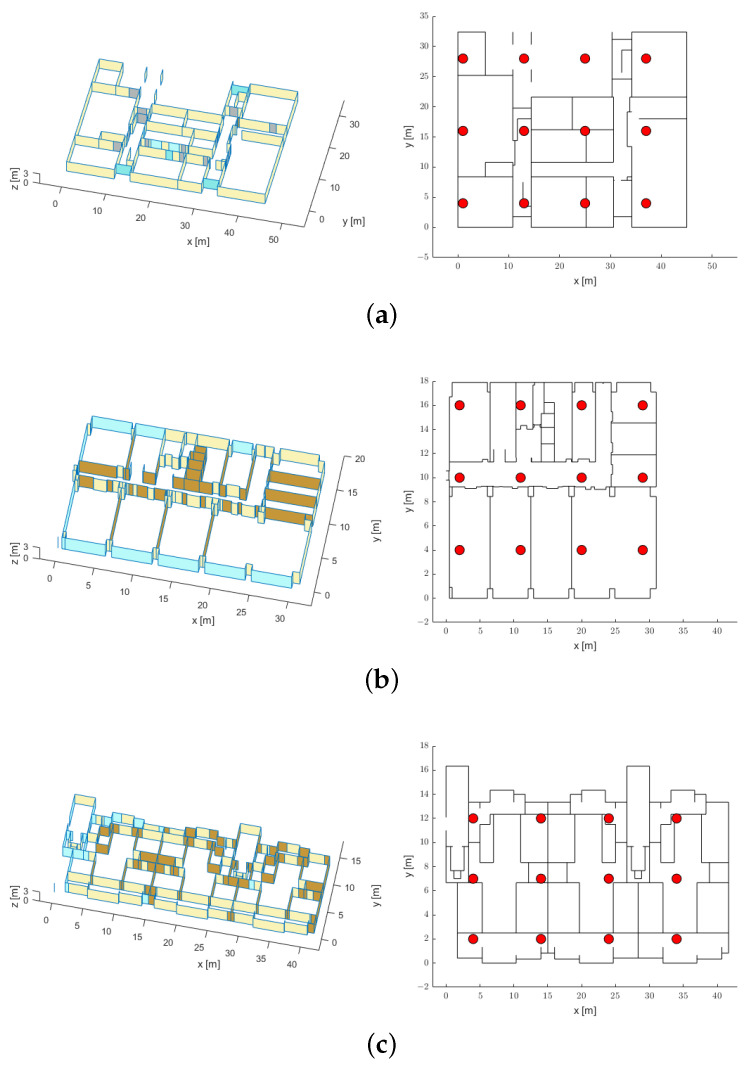
The 3D maps and the top views of the environments: (**a**) INMC (academic building), (**b**) ASRI (academic building), and (**c**) APT (residential space).

The proposed scheme assumes the installation of APs and the use of two frequency bands in an indoor environment. For the simulation, a geometry-based channel was created by using a ray-tracing method with real spatial information [46,47]. Three sites with different materials and structures were investigated. Figure 5 represents the 3D views and floor plans of the three sites, where (a) and (b) are academic buildings (INMC and ASRI on the Seoul National University campus), and (c) is an apartment building. The locations of the APs on each floor plan are marked with red circles. The height of the APs was set to 1.5 m for each map. The Tx power was assumed to be 0 dBm, and the center frequency was set to 2.4 or 5.2 GHz.

**Figure 6:**
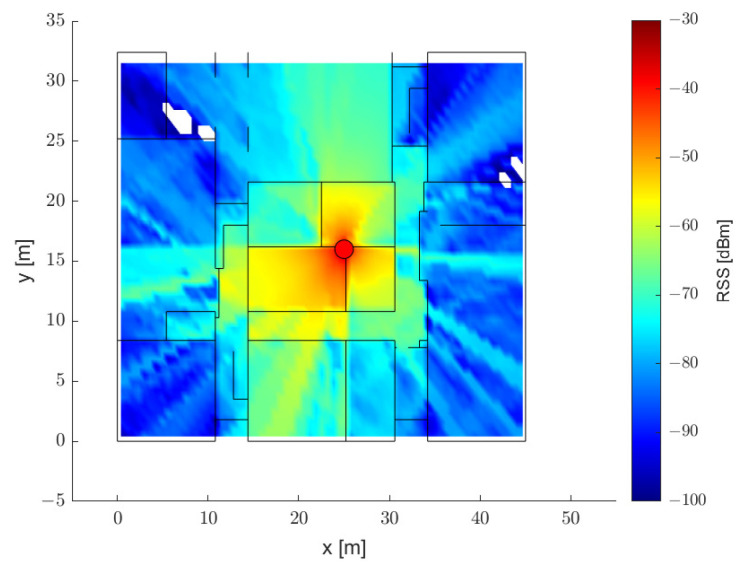
RSS map for an AP in the 2.4 GHz band.

**Figure 7:**
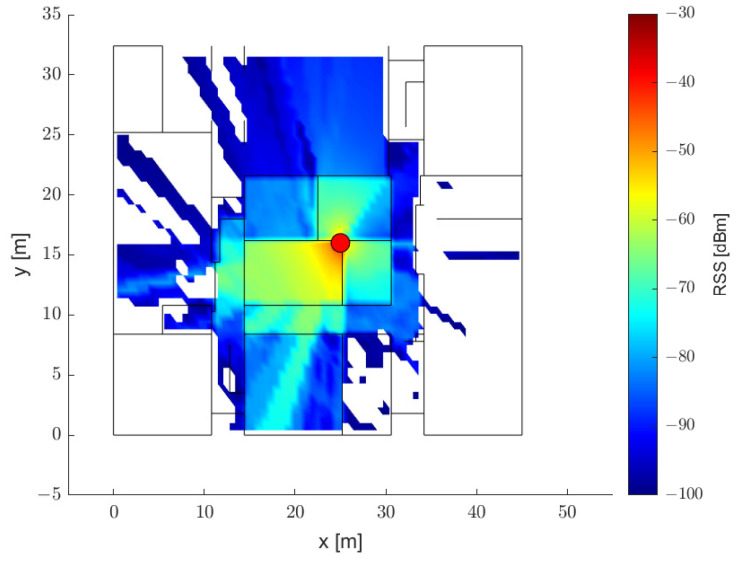
RSS map for an AP in the 5.2 GHz band.

Figure 6 and Figure 7 represent the RSS distribution according to the center frequency when only one specific AP is considered in the map shown in Figure 5a. Similarly to the theoretical inference, the attenuation in the 5.2 GHz band is larger than that in the 2.4 GHz band, and the difference in attenuation between the two frequency bands is verified to be larger under harsh NLOS conditions. For the evaluation of the ranging and positioning performance, the RSS values from 12 APs were recorded at points on a uniform grid approximately 1 m apart from each other for the three sites. The total number of points evaluated for the three sites was 11,474, and the number of RSS–distance pairs satisfying the noise floor condition was 74,912. All measurement points were split into sets with the following proportions: 70% for the training set, 15% for the validation set, and 15% for the test set; then, the deep regression model was trained and evaluated by using the dual-band RSS and distance pairs.

### 6.2. Analysis of the Ranging Accuracy

Several existing ranging methods were used as benchmarks to verify the effectiveness of the proposed method. Most previous RSS based ranging method used a single-band RSS, To analyze the effect of using dual-band RSS, benchmarks 1 and 2 were set as a rule-based raging method using only RSS in the 2.4 and 5.2 GHz bands, respectively. These were based on a two-slope PLM as described in (2) that has been widely used in existing studies. The PLM parameters for each frequency band were optimized with linear regression to best fit the training data. For a single RSS measurement, the distance can be estimated by PLM inversion as presented in (4). The ranging accuracy of these methods can be affected by the accuracy of the PLM models. To consider more complex PLM, benchmarks 4 and 5 used a non-linear regression model using a neural network for single-band RSS data. Similar to the proposed method, deep regression models were learned using the only RSS in the 2.4 and 5.2 GHz bands. The benchmark 3 is the result of an existing rule-based ranging algorithm that uses the dual-band RSS described in [34]. In the ranging method of Benchmark 3, the channel states were divided into LOS, NLOS, and severe NLOS, and the path-loss exponents were different for each state. The parameters of this rule-based methods were also optimized to best fit the training data.

**Figure 8:**
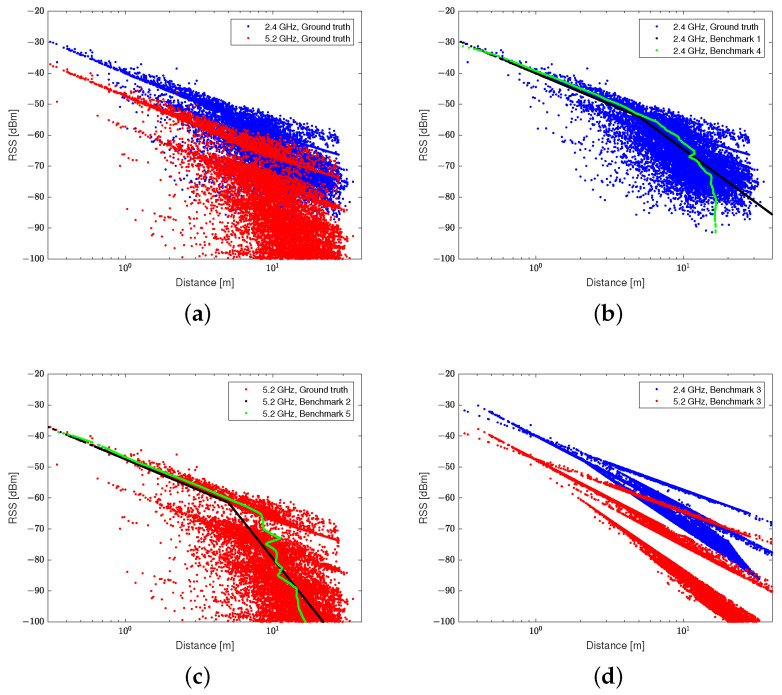
(**a**) Distance–RSS data of the ground truth, and the regression results of the (**b**) 2.4 GHz, (**c**) 5.2 GHz, and (**d**) dual-band frequencies.

In Figure 8, the results of benchmarks 1 and 2, which use the regression methods based on the two-slope PLM, are represented by black lines, and the regression model based on the neural network is expressed in green lines. The results in Figure 8b,c show the limitations of the ranging method using a single-band RSS. As only one distance is mapped to one RSS value, the NLOS conditions cannot be distinguished. The regression results of the two-slope PLM and the results of the neural network are similar when using the single-band RSS. The ranging method using a neural network shows an improvement in ranging accuracy. However, there is an upper limit for the ranging method using the single-band RSS, regardless of how detailed the tuning applied is. Figure 8d shows the ranging results of the existing rule-based ranging method using the dual-band RSS. In this method, two or more distances may correspond to the RSS value of one band. The distance can be estimated by classifying the NLOS conditions, but reflecting all of the various cases shown in (a) is difficult. In addition, because only the path-loss exponent is adjusted to estimate the distance for the NLOS conditions, there is a risk of amplifying the ranging error for misclassified cases.

**Figure 9:**
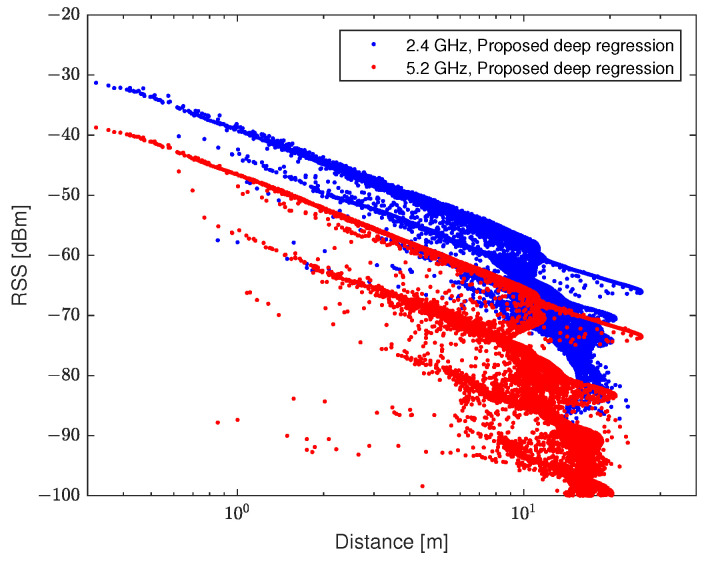
Regression results of the distance–RSS data with the proposed method.

The regression results of the proposed model are shown in Figure 9. The NLOS conditions were divided into several stages by using the dual-band RSS input. This reflects the severe NLOS conditions, especially when there are multiple walls between antennas. The deep regression model was trained using data from various cases. Therefore, it describes the ground truth of the test data better than the other rule-based models. In the performance evaluation, the number of nodes in the hidden block was set to 64, and the number of hidden blocks was set to 10.

**Figure 10:**
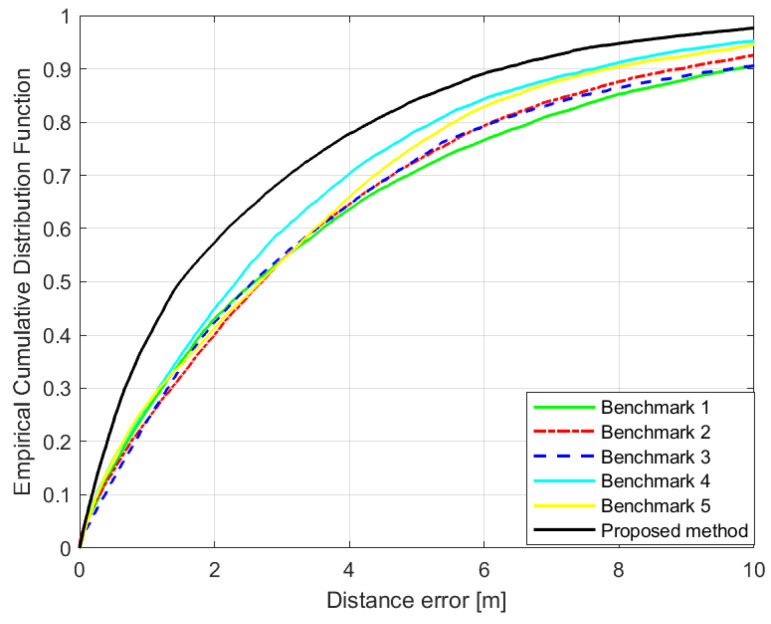
Empirical cumulative distribution function (ECDF) of the ranging error.

Figure 10 shows the empirical cumulative distribution function (ECDF) of the ranging errors according to the ranging methods. The key indicators of the results are summarized in Table 1. In terms of the error between the estimated distance and the actual distance, the proposed method showed the best performance with a median error of 1.49 m. This is approximately 36.1% lower than those of the benchmark techniques. The benchmark results using the single-band RSS showed similar performances. This is because there is a limit to the information that can be obtained from the single-band RSS. The results of Benchmark 3, which used dual-band RSS, did not show good ranging accuracy for the test environment. This is due to the limitation of the rule-based ranging method in that it is difficult to reflect various cases in an indoor environment with only three discrete channel states. Although the ranging accuracy improved in some cases, the statistical performance of the total test samples was degraded because ranging errors were amplified by misclassification in several other cases.

### 6.3. Analysis of the Neural Network Structure

**Figure 11:**
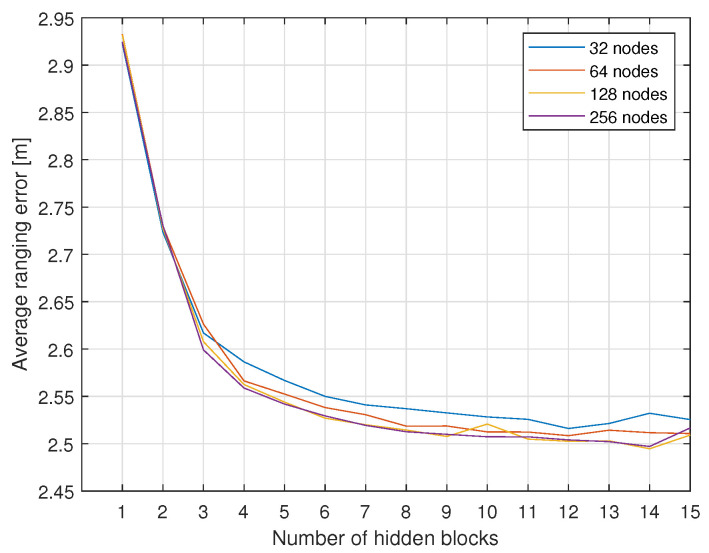
Average ranging error according to the structure of the neural network.

The hyperparameters of the proposed deep regression model are adjustable variables. There is a trade-off between the performance and computational complexity of the neural network. We evaluated the average value of the ranging error by changing the number of nodes and the number of hidden blocks, and Figure 11 shows the results. The ranging error is saturated to about 2.5 m, and having too many hidden blocks for the small number of nodes decreases the ranging accuracy. The computational complexity of the proposed model structure can be expressed as $O(M^{2}*L)$. Therefore, as the structure becomes more complex, the computation increases excessively compared to the performance gain. In this paper, we propose the use of 64 nodes in each layer and eight hidden blocks, where the ranging accuracy is greater than the 95th percentile value among the evaluated models, and the computational complexity is only 3.3% compared to the model with the highest ranging accuracy.

### 6.4. Analysis of Positioning Accuracy

**Figure 12:**
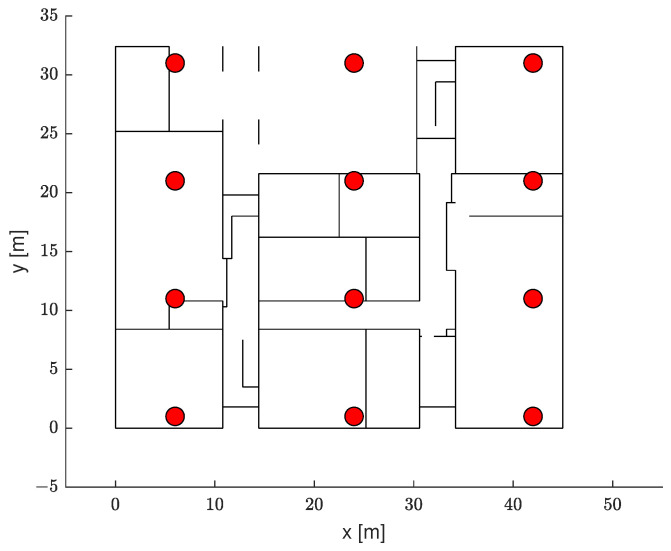
A top view of the test environment.

To analyze the effect of our proposed ranging technique on the range-based localization accuracy, we used the ILS positioning method discussed in Section 3. In particular, to reflect a change in the channel environment, we assumed a change in the AP deployment, as shown in Figure 12. The fingerprint-based methods cannot be applied without a new site survey, and the robustness of the proposed hybrid localization can be verified in this scenario.

**Figure 13:**
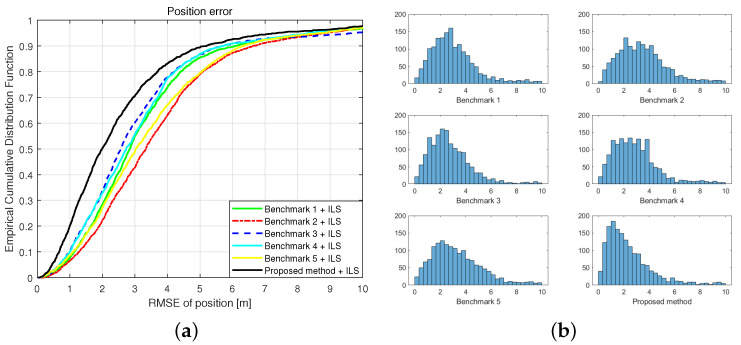
Empirical cumulative distribution function (**a**) and histogram (**b**) of the positioning error.

Figure 13a shows the ECDF of the root mean square error (RMSE) between the estimated and actual positions for a total of 1641 points. The key indicators are summarized in Table 2. In terms of statistical results, the performance of the proposed method was superior to that of the existing methods. The average RMSE of the proposed method is similar to that of Benchmark 1 due to the outlier result of the ILS positioning method. When comparing the median RMSEs, our proposed positioning method showed a lower error than those of other existing methods by at least 22.3%. Positioning results using the single-band RSS of 2.4 or 5.2 GHz had median errors of 2.73 m and 3.03 m, respectively, even when the deep regression method was applied. These are 3.8% and 9% smaller errors, compared to the results of using 2-slope PLM-based method for each band. Meanwhile, the result of benchmarks 3, which was the result of the existing rule-based method using the dual-band RSS, was 2.56 m. Additionally, the deep regression ranging method using the dual-band RSS could reduce the median of positioning error to 1.99 m. It was a 22.3% improved result compared to the result of benchmark 3. The effect of the deep regression method was more significant with the dual-band RSS than with the single-band RSS.

Our proposed method is more effective for errors of the 50th percentile or less. The histogram in Figure 13b shows the distribution of spatial positioning errors. In each histogram, the x-axis represents positioning RMSE, and the y-axis represents count. The result shows that proposed method improved the overall positioning accuracy, and increases the number of cases estimated within 2 m. This is because the proposed algorithm effectively reduces the ranging error when the AP and the target device are actually close to each other, but are blocked by walls.

## 7. Conclusions and Future Work

In this study, we proposed a hybrid localization algorithm that replaces only the ranging part of the existing range-based localization method with a deep regression model that uses data-driven learning. For the ranging part, the accuracy of distance estimation in an indoor NLOS environment is improved by using the dual-band RSS, and this is designed to cover various cases by using regression with a neural network. The improvements in the ranging and positioning accuracy of the proposed method were verified through a ray-tracing-based simulation for general indoor spaces. Furthermore, the proposed localization method is compatible with various situations, even when the indoor structure is changed. The prevailing fingerprint-based methods are cannot be applied in the situation we assumed where the deployment of APs are different compared to the offline stage.

Based on the results of ray-tracing based simulation, the use of dual-band RSS could reduce the median of positioning error up to 2.56 m, which was a 9.9% smaller error than the error obtained with the single-band RSS alone in the rule-based localization. By applying the deep regression method to the ranging part, the median error of 1.99 m was obtained, which was a 22.3% improved result compared to the rule-based method. Considering that the use of deep regression with single-band RSS only caused 10% or less performance gain, the combination of the dual-band RSS and the deep regression method was effective in improving the accuracy of range-based localization using RSS.

The robustness of the proposed method encourages the use of RSS localization because it can reduce the intensity of the site survey process. In addition, the proposed method has the advantage that it can be implemented by using only the beacon signals from APs, without any special communication overhead. This will be useful for small IoT devices that are difficult to mount with multiple types of sensors or high cost network interface controller.

However, some future research should be followed to apply the proposed method in various situations. In this study, we considered the wall structure as a major factor of the complex indoor channel. In the real environment, there are many small obstacles that cannot be reflected in the ray-tracing based simulation, and the RSS fluctuations caused by the multi-path effect. In addition, analysis of more diverse materials and interior structures is required. After all, for a deep regression ranging model that operates robustly in various environments, as much experiments as possible are required to train and to validate the model.

Nevertheless, the proposed method is still promising in that a properly trained ranging model can be used even in an untrained environment. In practice, we expect to increase the learning efficiency by using a transfer learning technique that tunes the model trained through simulation with relatively small dataset from real experiments. Furthermore, extending the research to the triple-band-based ranging method is possible by utilizing 6 GHz band communication, which is expected to be supported in the Wi-Fi 6E standard. 

## Figures and Tables

**Table 1:** Ranging error results of each ranging method.

Ranging Method	Ranging Error [m]			
	25%-Tile	50%-Tile	90%-Tile	Average
Benchmark1	0.96	2.60	9.75	4.05
Benchmark2	1.06	2.71	8.92	3.78
Benchmark3	1.05	2.57	9.67	4.580
Benchmark4	0.903	2.33	7.65	3.23
Benchmark5	0.88	2.68	7.85	3.471
Proposed method	0.53	1.49	6.28	2.52

**Table 2:** Positioning error results for each ranging method.

Ranging Method	Positioning Error [m]			
	25%-Tile	50%-Tile	90%-Tile	Average
Benchmark1	1.85	2.84	6.09	3.82
Benchmark2	2.11	3.33	6.63	4.98
Benchmark3	1.66	2.56	5.69	11.38
Benchmark4	1.67	2.73	5.70	28.38
Benchmark5	1.93	3.03	6.33	4.82
Proposed method	1.14	1.99	5.19	3.68
